# Scott’s Emulsion of Cod-Liver Oil in Eye and Laryngeal Troubles

**Published:** 1898-09

**Authors:** A. Bethune Patterson

**Affiliations:** Atlanta, Ga.


### SCOTT'S EMULSION OF COD-LIVER OIL IN EYE AND LARYNGEAL TROUBLES.

By A. BETHUNE PATTERSON, M.D.,

Atlanta, Ga.

Many eye and throat troubles that we are called upon to treat, and which appear to be local maladies, really have their origin in some *materies morbi* circulating in the blood. Their successful management depends largely upon our recognition of the peculiar constitutional conditions; then, again, there are local troubles, chronic in charter, that do not appear to be due to any special constitutional dyscrasia, though make little progress to recovery when only local remedies are applied, but quickly yield to a course of tonics, alteratives, and remedies which directly improve the nutritive fluids.

There are a host of therapeutic agents which are classified as tonics and alteratives, and whose therapeutic action are unexplainable, but exert a salutary influence on the nutrition, either directly or indirectly. Of the former there are but few. Cod-liver oil I regard as preeminently the most fitting. It is not only a food agent, but possesses medicinal properties as well. Its antiseptic properties either exert a retarding influence upon germ life, or antidotal or nutritizing effect upon the toxins. Lard and other oils have long enjoyed the reputation of possessing antidotal properties in cases of poisoning. It is a well-know fact that a fat hog suffers no inconvenience from the bite of a rattlesnake, while a lean one quickly succumbs. Melted lard is a domestic remedy in all cases of poisoning in domestic animals. The popularity that castor-oil enjoys is surely not entirely due to its cathartic or laxative effect. That it has a controlling influence on the ferments in the alimentary canal there can be no doubt.

The medical properties of cod-liver oil do not appear to be appreciated generally. It increases the appetite, and with it the digestion improves, the bowels become regular, there is a marked increase in the red corpuscles of the blood, the cheeks of the pale and anemic become rosy, the ears, lose their waxy appearance and become plethoric. The oil has a wonderful tonic effect upon cells which are rapidly exfoliating and therefore never reach a state of maturity or full growth.

It is an erroneous idea to suppose that cod-liver oil is a remedy for consumption only. It is a remedy peculiarly fitted to meeting the indications in nearly all chronic ailments. Tne unpalatableness has been its greatest drawback, though this has been largely overcome in the preparation known as Scott's Emulsion, containing the hypophosphites, which are valuable adjuncts to the alterative and antiseptic properties. I have always prepared the oil in an emulsion.

Shoemaker says: "There can be no question that the digestibility of the oil is increased by mechanical and chemical conditions, as when in the form of a good emulsion, . . . and by the addition of pancreatin, and also by the association with certain restorative agents like the hypophosphites."

The following cases from my note-book illustrate the prompt action of cod-liver oil in combination with the hypophosphites:

C. M., aged 9, comes on account of corneal ulcers. With the exception of repeated attacks of these ulcers during the last three years the general health has been considered good. The history shows that the ulcers were obstinate and did not yield to the iron tonics and local treatment in a satisfactory manner. This attack is of four week's duration; the general health has suffered, spirits depressed, appetite and digestion poor, bowels constipated. Ordered,

℞ Hydrarg chl. mit ........................................................ gr. i.
Santonine ........................................................ gr. ss.
Sugar milk, q. s. to make one powder.
To be given at bedtime, and repeat if necessary next morning.

Also,

℞ Pure cod-liver oil, sterilized........................................ ℥ i.
Atropine........................................................ gr i.
M. S. Drop in eyes every three or four hours, and to be bathed frequently with antiseptic lotion of boracic acid. Scott's Emulsion of cod-liver oil in teaspoonful doses three or four times a day.

The improvement was observed by parents as being more rapid than in former attacks when iron formed the basis of constitutional treatment.

G. R., aged 6, comes with phlyctenular ophthalmia, with history of subsequent attacks; tongue furred, disordered digestion, abdomen distended and tympanitic, ears waxy, indicating poor blood. Ordered hydrarg. chl. mit. and santonine, half-grain doses night and morning until bowels are freely moved.

℞ Atropin........................................................ gr. i.
Fluid ext. hydrastin........................................................ m x.
Glycerine........................................................ m xx.
Aqua........................................................ ℥ i.
M. S. Drop in eyes three or four times a day. Teaspoonful doses of Scott's Emulsion of cod-liver oil three times a day.

At the end of three weeks patient discharged with instructions to continue the cod-liver oil three weeks longer.

J. W., aged 30. Chronic naso-pharyngeal catarrh and chronic laryngitis. Comes on account of the latter trouble, which is of two years' standing; voice is husky, and complains of pain and fullness in larynx; cough, with some expectoration, which is free of tubercle bacilli; a constant feeling of languor and debility. After three months' treatment, consisting of daily local applications to larynx and naso-pharynx, with Scott's Emulsion of Cod-liver Oil three times a day, and laxatives, with the improvement of health and laryngitis, his visits were discontinued to my office.

*507 English-American Building.*

